# Cancer risk in Korean patients with Behçet’s disease: A nationwide population-based study

**DOI:** 10.1371/journal.pone.0190182

**Published:** 2017-12-29

**Authors:** Yoon Suk Jung, Minkyung Han, Do Young Kim, Jae Hee Cheon, Sohee Park

**Affiliations:** 1 Division of Gastroenterology, Department of Internal Medicine, Kangbuk Samsung Hospital, Sungkyunkwan University School of Medicine, Seoul, Republic of Korea; 2 Department of Public Health, Graduate School, Yonsei University, Seoul, Republic of Korea; 3 Department of Dermatology and Cutaneous Biology Research Institute, Yonsei University College of Medicine, Seoul, Republic of Korea; 4 Department of Internal Medicine and Institute of Gastroenterology, Yonsei University College of Medicine, Seoul, Republic of Korea; 5 Department of Biostatistics, Graduate School of Public Health, Yonsei University, Seoul, Republic of Korea; Oregon Health and Science University, UNITED STATES

## Abstract

**Background:**

Various immune-mediated diseases are associated with increased malignancy risks. However, the relationship between Behçet’s disease (BD) and cancer remains unclear. We conducted a nationwide, population-based study to determine the risk of cancer in patients with BD.

**Methods:**

Using National Health Insurance claims records, we collected data from 2402 patients diagnosed with BD between 2013 and 2014. Standardized incidence ratios (SIRs) of overall and site-specific cancers in patients with BD in comparison with the general population were calculated.

**Results:**

The risks of overall cancer (SIR, 3.54; 95% confidence interval, 2.35–5.11 in men and 2.17; 1.58–2.92 in women) and solid cancer (3.10; 1.94–4.69 in men and 2.13; 1.52–2.90 in women) were greater in patients with BD than in the general population. There were significantly increased risks for these solid cancers: colorectal (4.26; 1.38–9.94), liver (4.00; 1.09–10.25), bone/articular cartilage (55.66; 1.41–310.14), prostate (7.05; 1.45–20.60), and brain/central nervous system (28.32; 3.43–102.31) in men; and the lips/oral cavity/pharynx (13.97, 1.69–50.47), liver (12.78; 5.14–26.33), lungs (4.35; 1.18–11.13), other female genital organs (53.57; 1.36–298.49), and eyes (128.26; 3.24–714.59) in women. Patients with BD had a greater risk of myelodysplastic syndrome (MDS) than the general population did (65.72; 7.96–237.41 in men and 53.86; 11.11–157.40 in women), but not of hematological cancer.

**Conclusions:**

Compared to the general population, Korean patients with BD had greater risks of overall cancer, some solid cancers, and MDS, but not of hematological cancer.

## Introduction

Behçet’s disease (BD) is a chronic, multisystemic, immune-mediated disorder that is characterized by recurrent oral and/or genital ulcers, arthritis, and skin manifestations, as well as ocular, vascular, neurological, or intestinal involvement [1,2]. Autoimmunity and chronic inflammation are associated with malignancy development [3–5]. Immune-mediated diseases, which result from a dysregulated immune response, often cause chronic inflammation [3,4]. Chronic and unregulated inflammation can cause malignant cell transformation and carcinogenesis through inflammation-related mechanisms [3,4]. Chronic exposure of inflammatory mediators, including metabolites of arachidonic acids, cytokines, chemokines, and free radicals, leads to increased cell proliferation, mutagenesis, oncogene activation, and angiogenesis [3,4].

Several epidemiological studies have evaluated the relationships between a variety of systemic autoimmune rheumatic diseases and cancer, and the studies have demonstrated that rheumatoid arthritis (RA), systemic lupus erythematosus (SLE), Sjögren’s syndrome, systemic sclerosis, and dermatomyositis, are associated with an increased risk of malignancy, especially lymphoma [5–9]. BD has also been reported to be sporadically associated with malignancy [10,11]. In particular, BD has been reported to be associated with hematological cancers in addition to bone marrow failure, such as myelodysplastic syndrome (MDS) and aplastic anemia (AA) [10–13]. Given that organs commonly involved in BD include the oral cavity, genitalia, skin, and eye and that BD recurrently induces chronic inflammation of these organs, BD may also confer a high risk of cancer in these organs. However, data on the relationship between BD and solid cancers are very scarce. Moreover, most previous studies investigating the associations between BD and cancer were case reports or hospital-based chart reviews with inconsistent results [10,11,14]. Thus, based on current evidence, it is difficult to conclude that BD itself increases the risk of cancer development.

To clarify the relationship between BD and cancer risk, we conducted a nationwide, population-based study to compare the overall and site-specific cancer risks in patients with BD against those in the general population using national insurance claims data in Korea.

## Material and methods

### Data source

As the Korean government operates a mandatory nationwide insurance system (National Health Insurance; NHI), all health care utilization information is registered under a comprehensive database operated by the Health Insurance and Review Agency (HIRA). Using the HIRA database allowed us to provide estimates based on the occurrence of events in unselected patient populations representing the whole spectrum of disease extent and severity.

This study used data from the NHI, which provides mandatory universal health insurance that covers all forms of health services, including hospitalization, ambulatory care, and pharmaceutical services. Medical institutions submit health care utilization information in electronic format for reimbursement purposes, and this information is integrated into the HIRA claims database, which covers the entire population of Korea (about 51 million people). The database contains information on all patients, including demographic characteristics, ambulatory care history, principal diagnosis and comorbidities using the International Classification of Diseases, 10th revision (ICD-10), prescriptions, and procedures.

Beginning in 2006, the NHI initiated a registration program for 138 rare intractable diseases (RIDs), including BD, in order to subsidize the medical expenses of patients with RIDs. Through the RID program, the Korean government covers 90% of the hospital expenses for patients with RIDs so that those patients pay for only 10% of their hospital expenses. Considering these medical benefits, a diagnosis of BD is not likely to be missed in the RID system. Additionally, the diagnosis must be made based on very strict diagnostic criteria provided by the RID system and must be reviewed by the corresponding healthcare institution before being submitted to the NHI. Thus, data regarding RIDs are verified and reliable. Our study only included cases of BD certified as RIDs in the HIRA claims database.

### Patient identification

Patients identified as having BD in HIRA from January 2011 to December 2015 were included in this study. In the RID program, BD was diagnosed using the International Study Group diagnostic criteria for BD (recurrent oral ulcerations plus two of the following criteria: recurrent genital ulcerations, eye lesions, skin lesions, and positive pathergy test) [2]. To increase the diagnostic accuracy of BD, BD was determined in cases that met both the appropriate diagnostic code (ICD-10 code: M35.2) and the RID code (V139). The date of initial registration in the RID program during the study period was regarded as the date of BD diagnosis.

### Ascertainment of cancer incidence rates

Cancer occurrence in BD patients was ascertained using the NHI database. For the comparison with the cancer incidence rates in the general population, we used the national database of The Korea Central Cancer Registry (KCCR) that covers the entire population in Korea [15]. Besides all incident cases of cancer (ICD-10: C00-C96), we also investigated the number of incident cases of MDS (ICD-10: D46) and AA (ICD-10: D61) because several previous studies have reported that BD is associated with these hematological diseases [10–12].

### Statistical analysis

The source population for this study consisted of all patients with NHI claims data between 2011 and 2015. Because the inclusion of previous prevalent cases may confound the incidence, we applied a washout period of 2 years by excluding the patients that had any claim record for BD during these 2 years in recognition of these cases as prevalent rather than incident cases. To ensure accurate diagnosis, an incident case for BD was defined as a patient registered for BD for 2 years in a row. Thus, we could assess the incidence of BD between 2013 and 2014. The incidence rate was defined as the number of incident cases in the corresponding year per 100,000 people using the mid-year population size (resident registration population on July 1 of each year). Age and sex-specific incidence rates were calculated by dividing the number of cases in age- and sex-stratified groups by the corresponding age- and sex-specific population and was expressed as cases per 100,000 people.

To determine whether patients with BD had a greater risk of developing cancer than did the general population, we calculated the standardized incidence ratio (SIR, a ratio of observed to expected cancers) of overall and site-specific cancers in patients with BD. The number of observed cancer cases used only newly diagnosed cancer cases. The KCCR provides information on the general population’s incidence rate of cancer in 10-year age intervals, from age 0 to 99 years. Using the sex- and age-specific incidence rates of the general population from KCCR data, the observed and expected numbers of cases in BD patients were calculated by sex- and age-specific groups. The number of expected cancer cases was calculated by multiplying the age-specific cancer incidence rate of the general population from 2013 and the person-years of patients with BD. The 95% confidence intervals (CIs) for the SIRs were calculated using Poisson’s distribution.

To investigate the association between medication and the risk of cancer incidence, a nested case control analysis was used, which would avoid the length bias produced by a regular case control analysis and would match exposure durations of cases and controls. We also matched the diagnosis dates of BD to avoid temporal bias. For each subject who developed cancer during the follow-up period, four cancer-free subjects were randomly selected and matched to each cancer case with the same sex, age and diagnosis date of BD. We then examined whether the cases and controls used medications during the period beginning with the diagnosis of BD and ending with each case’s respective cancer diagnosis date (for controls, their matching case’s respective cancer diagnosis date). Taking into account the matched case control data structure, we used conditional logistic regression to estimate the OR and corresponding 95% CIs. SAS Enterprise Guide [SAS Institute, Inc.] was used for all statistical analyses. P values less than 0.05 were considered statistically significant.

### Ethical considerations

All identifiable personal information in medical records was de-identified to comply with the privacy rule of the Health Insurance Portability and Accountability Act. In addition, as the information in the HIRA database is encrypted, the database does not contain personal identifiers. This study protocol was approved by the Institutional Review Board of Severance Hospital, Yonsei University College of Medicine (IRB No.4-2016-1135).

## Results

### Study population and incidence

The baseline characteristics of the study population are presented in Table 1. Overall, 2402 patients with BD were newly diagnosed between 2013 and 2014. The mean age at diagnosis was 44.5 ± 13.1 years, and the male-to-female ratio was 0.54. The proportion of patients diagnosed with BD was highest in the 40–49 year age group (29.4%). Among all incident cases, 2178 (90.7%), 1,986 (82.7%), 373 (15.5%), 599 (24.9%), 662 (27.6%), and 30 (1.2%) were prescribed corticosteroids, colchicine, sulfasalazine, 5-aminosalicylic acid (ASA), thiopurine, and tumor necrosis factor alpha (TNF-α) inhibitors (infliximab or adalimumab) during the follow-up period. Of the 30 patient who received TNF-α inhibitors, 13 (0.5%) were treated using infliximab, and 19 (0.8%) were treated using adalimumab.

**Table 1:** Baseline characteristics of the study population.

Characteristic	N (%)
Total population	2402
Men	847 (35.3)
Women	1555 (64.7)
Age at diagnosis (years; mean ± SD)	44.5 ± 13.1
<20 years	72 (3.0)
20–29 years	246 (10.2)
30–39 years	500 (20.8)
40–49 years	705 (29.4)
50–59 years	614 (25.6)
60–69 years	198 (8.2)
≥70 years	67 (2.8)
Medication use	
Corticosteroid	2,178 (90.7)
Colchicine	1,986 (82.7)
Sulfasalazine	373 (15.5)
5-ASA	599 (24.9)
Thiopurines	662 (27.6)
TNF- α inhibitors	30 (1.2)
Infliximab	13 (0.5)
Adalimumab	19 (0.8)

SD, standard deviation; 5-ASA, 5-aminosalicylic acid; TNF-α, tumor necrosis factor alpha

**Fig 1:**
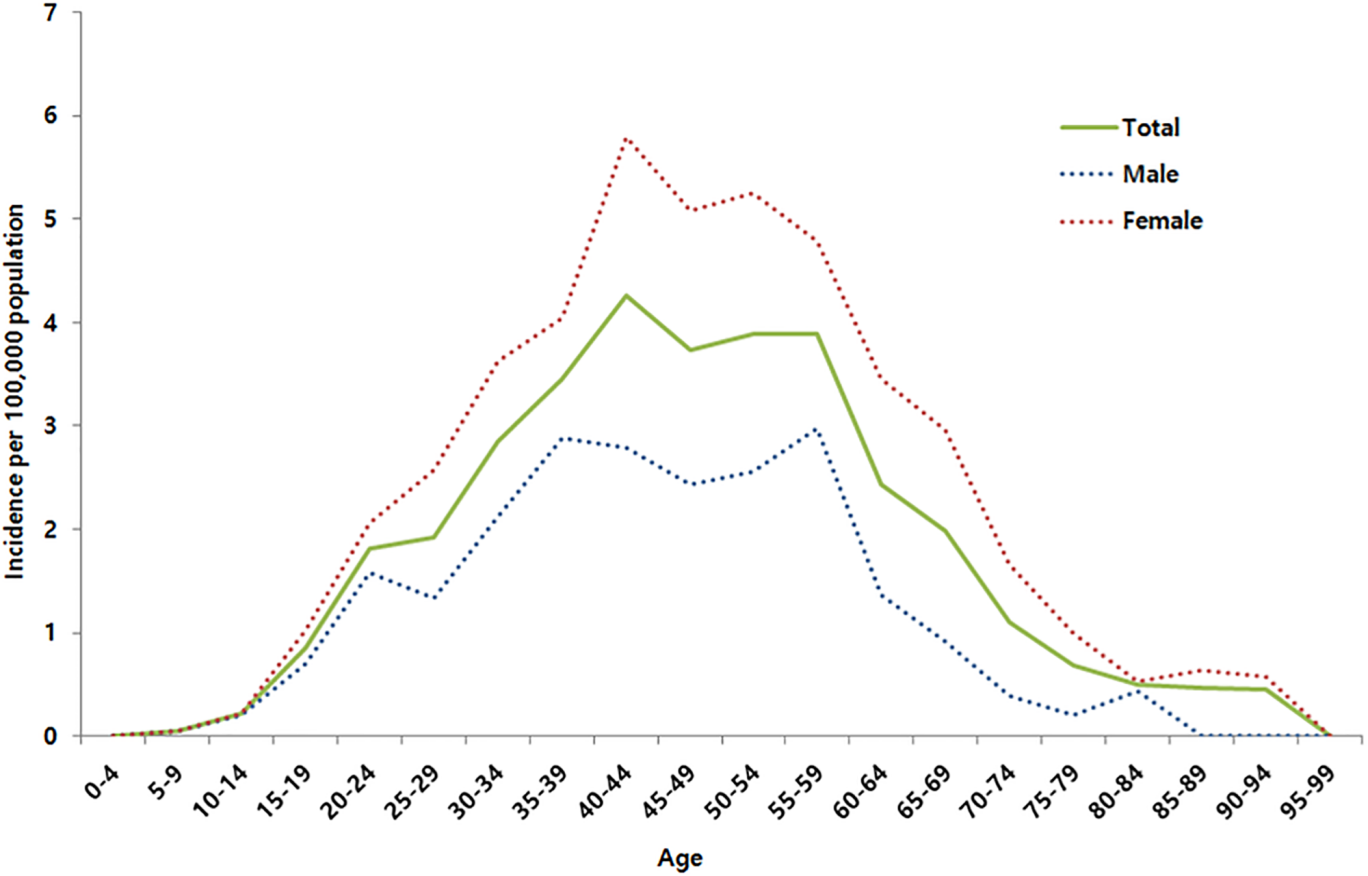
Incidence of Behçet’s disease by sex and age group from 2013 to 2014.

The incidence of BD by sex and age group is shown in Fig 1. The incidences of BD in 2013 and 2014 were 2.44 and 2.30 per 100,000 person-years, respectively, with an average annual incidence of 2.37 per 100,000 person-years. The average annual incidences of BD in men and women were 1.67 and 3.07 per 100,000 person-years, respectively. The incidence of BD was highest in the 40–44 year age group (4.27 per 100,000 person-years) and steadily decreased thereafter.

### Cancer risk in patients with Behçet’s disease

**Fig 2:**
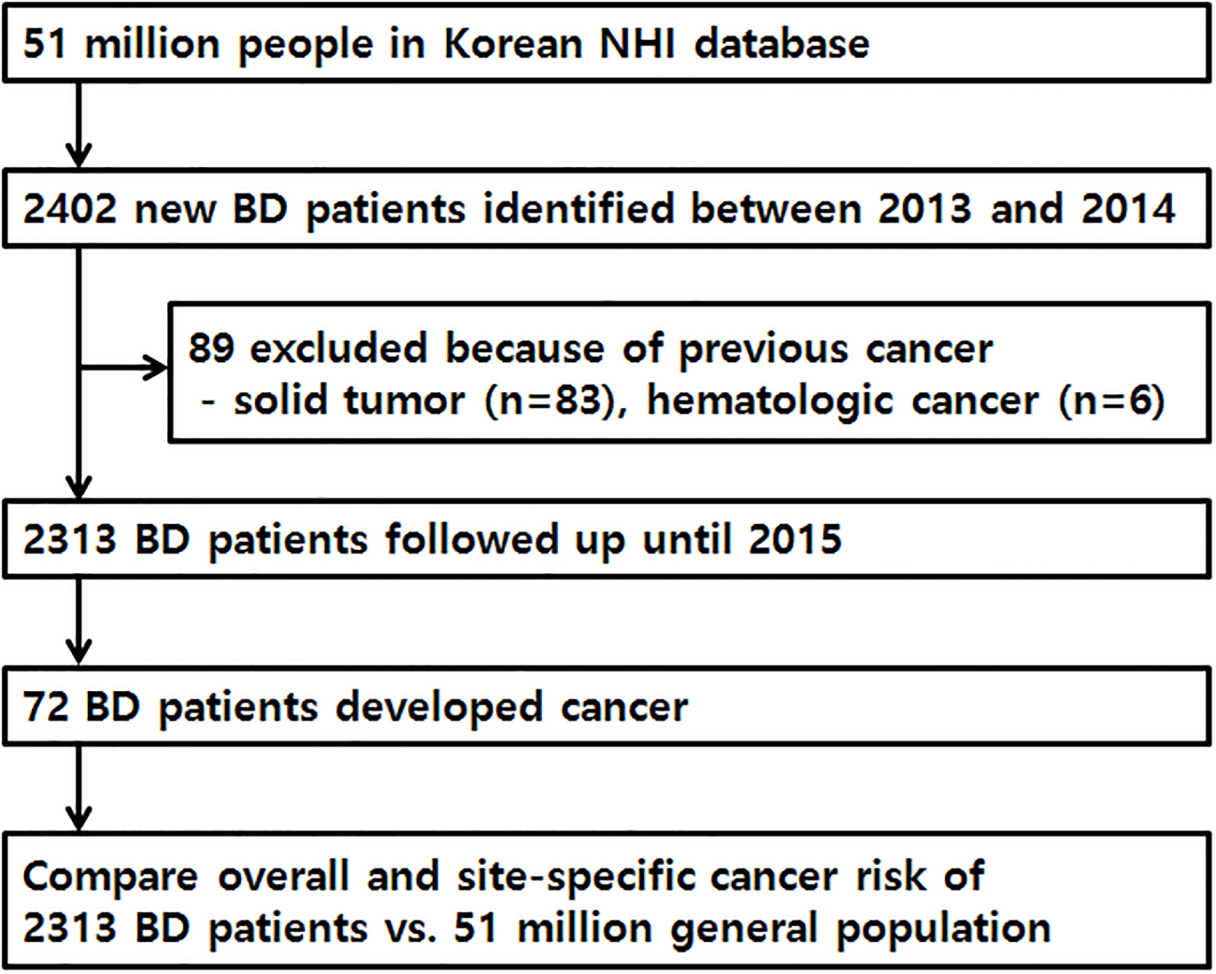
Flow chart of the study process.

After excluding 89 patients with previous cancer, a total of 2313 patients with BD were included in the cancer SIR analysis and followed-up until December 31, 2015. The 89 excluded patients with previous cancers had 6 hematological cancers and 83 solid cancers (Fig 2). The 2313 patients with BD were followed for 5854 person-years (median follow-up duration, 2.34 years; range, 0.12–4.99 years).

The SIRs of overall and site-specific cancers in patients with BD are presented in Table 2. Among the 823 men with BD, 28 cancers were observed (vs. 7.91 expected), which resulted in an increased overall cancer risk compared to that in the general population (SIR, 3.54; 95% CI, 2.35–5.11). Compared with those in the general population, men with BD had significantly increased risks of colorectal cancer (CRC) (SIR, 4.26; 95% CI, 1.38–9.94), liver cancer (SIR, 4.00; 95% CI, 1.09–10.25), bone/articular cartilage cancer (SIR, 55.66; 95% CI, 1.41–310.14), prostate cancer (SIR, 7.05; 95% CI, 1.45–20.60), and brain/central nervous system (CNS) cancer (SIR, 28.32; 95% CI, 3.43–102.31). Overall, 22 solid cancers were observed (vs. 7.11 expected), resulting in an increased overall solid cancer risk (SIR 3.10; 95% CI 1.94–4.69). On the other hand, the risk of hematological cancer was not significantly higher in men with BD (SIR, 2.58; 95% CI, 0.07–14.35); only one man with BD developed multiple myeloma vs. 0.04 expected (SIR, 22.44; 95% CI, 0.57–125.05).

**Table 2:** Risks of site-specific cancer in patients with Behçet’s disease.

Cancer (ICD-10 code)	Men			Women		
	Observed, n	Expected, n	SIR (95%CI)	Observed, n	Expected, n	SIR (95%CI)
All cancer (C00–C96)	**28**	**7.91**	**3.54 (2.35–5.11)**	**44**	**20.24**	**2.17 (1.58–2.92)**
Solid cancer	**22**	**7.11**	**3.10 (1.94–4.69)**	**40**	**18.79**	**2.13 (1.52–2.90)**
Lip, oral cavity, and pharynx (C00–C14)	-			**2**	**0.14**	**13.97 (1.69–50.47)**
Stomach (C16)	3	1.48	2.03 (0.42–5.93)	4	1.50	2.67 (0.73–6.82)
Colon and rectum (C18–C20)	**5**	**1.17**	**4.26 (1.38–9.94)**	2	1.61	1.24 (0.15–4.49)
Liver (C22)	**4**	**1.00**	**4.00 (1.09–10.25)**	**7**	**0.55**	**12.78 (5.14–26.33)**
Gallbladder (C23–C24)	-			1	0.27	3.69 (0.09–20.54)
Pancreas (C25)	1	0.19	5.39 (0.14–30.03)	1	0.29	3.40 (0.09–18.94)
Lung (C33–C34)	1	0.82	1.22 (0.03–6.81)	**4**	**0.92**	**4.35 (1.18–11.13)**
Bone and articular cartilage (C40–C41)	**1**	**0.02**	**55.66 (1.41–310.14)**	-		
Skin (C43–C44)	1	0.13	7.95 (0.20–44.29)	1	0.28	3.55 (0.09–19.78)
Breast (C50)	-			3	3.90	0.77 (0.16–2.25)
Cervix, uterus (C53)	-			2	0.71	2.80 (0.34–10.12)
Other female genital organ (C57)	**-**			**1**	**0.02**	**53.57 (1.36–298.49)**
Prostate (C61)	**3**	**0.43**	**7.05 (1.45–20.60)**	-		
Kidney (C64)	-			1	0.23	4.41 (0.11–24.55)
Eye (C69)	**-**			**1**	**0.01**	**128.26 (3.24–714.59)**
Brain and CNS (C70–C72)	**2**	**0.07**	**28.32 (3.43–102.31)**	1	0.13	7.50 (0.19–41.80)
Thyroid (C73)	1	0.95	1.05 (0.03–5.86)	9	7.45	1.21 (0.55–2.29)
Hematological cancer	1	0.39	2.58 (0.07–14.35)			
Multiple myeloma (C90)	1	0.04	22.44 (0.57–125.05)	-		
Other (remaining cancer codes)	**5**	**0.42**	**11.89 (3.86–27.75)**	**4**	**0.82**	**4.89 (1.33–12.52)**

ICD-10, International Classification of Diseases, 10^th^ revision; SIR, standardized incidence ratio; CI, confidence interval, CNS, central nervous system.

Bold text in this table indicates statistically significant results.

Among the 1490 women with BD, 44 cancers were observed (vs. 20.24 expected), which resulted in an increased overall cancer risk compared to that in the general population (SIR, 2.17; 95% CI, 1.58–2.92). Similar to men with BD, women with BD had a significantly increased risk of overall solid cancer, but not of hematological cancer. Women with BD had increased risks of lip/oral cavity/pharynx cancer (SIR, 13.97; 95% CI, 1.69–50.47), liver cancer (SIR, 12.78; 95% CI, 5.14–26.33), lung cancer (SIR, 4.35; 95% CI, 1.18–11.13), other female genital organ cancer (SIR, 53.57; 95% CI, 1.36–298.49), and eye cancer (SIR, 128.26; 95% CI, 3.24–714.59). Overall, 40 solid cancers were observed (vs. 18.79 expected), resulting in an increased overall solid cancer risk (SIR, 2.13; 95% CI 1.52–2.90). However, none of women with BD developed hematological cancer.

Because several studies have reported that BD is associated with MDS and AA [10–12], we further investigated the risk of these diseases in patients with BD in the present study. Two men and three women developed MDS; it followed that patients with BD had a significantly greater risk of MDS than the general population did (vs. 0.03 expected; SIR, 65.72; 95% CI, 7.96–237.41 in men and vs. 0.06 expected; SIR, 53.86; 95% CI, 11.11–157.40 in women). The SIR of AA in patients with BD was not calculated since the incidence rate of AA in the general population was not available; nonetheless, we identified six patients (two men and four women) who developed AA.

### Risk of cancer according to medication use

The risk of solid cancer and overall cancer was lower in ever users of thiopurines than in never users of this drug (OR, 0.28; 95% CI, 0.10–0.77 and 0.35; 0.14–0.86, respectively). However, the use of sulfasalazine or 5-ASA was not associated with the risk of solid cancer (OR, 1.51; 95% CI, 0.72–3.15) or overall cancer (OR, 1.81; 95% CI, 0.93–3.53) (Table 3). Because none with solid cancer or overall cancer used TNF-α inhibitors, we could not assess their association. Also, because only one man with BD developed hematological cancer, we could not assess the risk of hematological cancer according to medication use.

**Table 3:** Risk of cancer according to medication use in patients with Behçet’s disease.

	Solid cancer OR	Overall cancer OR
Sulfasalazine or 5-ASA ever use		
No	1 (Reference)	1 (Reference)
Yes	1.51 (0.72–3.15)	1.81 (0.93–3.53)
Thiopurines ever use		
No	1 (Reference)	1 (Reference)
Yes	**0.28 (0.10–0.77)**	**0.35 (0.14–0.86)**

5-ASA, 5-aminosalicylic acid; OR, odds ratio

Bold text in this table indicates statistically significant results.

## Discussion

In this first Korean population-based analysis of cancer risk in patients with BD, we found that patients (both men and women) with BD had greater risks of overall cancer and solid cancer compared to those in the general population. Men with BD had an increased risk of CRC, liver cancer, bone/articular cartilage cancer, prostate cancer, and brain/CNS cancer. Women with BD had an increased risk of lip/oral cavity/pharynx cancer, liver cancer, lung cancer, other female genital organ cancer, and eye cancer. Patients (both men and women) with BD also had a greater risk of MDS than did the general population. However, the risk of hematological cancer in patients with BD was not greater than that in the general population.

To date, few population-based studies have been conducted to determine the cancer risk among patients with BD. Only two recent studies have investigated the cancer risk in patients with BD at the population level in Taiwan [16,17]. Similar to our results, the Taiwan study of 1314 new BD patients identified between 2000 and 2009 demonstrated that patients with BD had a greater risk of overall cancer (SIR 1.5, 95% CI 1.03–2.1) [16].

Given that BD is a chronic, multisystemic, immune-mediated disorder that is characterized by oral, genital, skin, ocular, intestinal, and/or neurological involvement, it is interesting, but not surprising, that we found that patients with BD had a greater risk of solid cancer than did the general population. The increased risk in patients with BD was particularly evident for solid cancers in organs that can be involved in BD (such as the colon and rectum, articular cartilage, and brain/CNS in men and the lips/oral cavity/pharynx, female genital organ, and eyes in women). A dysregulated immune response can cause chronic inflammation, which may in turn, through inflammatory mediators, cause malignant cell transformation and carcinogenesis in involved organs [3,4]. Several studies have reported a relationship between BD and hematologic cancers or diseases [10–13], whereas research on the association of BD with the risk of solid cancer has been rare. A Chinese study involving 41 BD patients who developed malignancies demonstrated that CRC was the most common solid tumor associated with BD [10]. A Korean study reported that of 1769 patients with BD, 21 (1.2%) developed solid cancer, and thyroid cancer was the most common solid cancer occurring in patients with BD [11]. However, these two studies simply presented descriptive statistics for the most common solid cancers among patients with BD; thus, based on their results, it was difficult to determine whether patients with BD had a greater risk of those solid cancers than did people in the general population. To date, only one study has reported an increased risk of certain solid cancers in patients with BD at the population level. A Taiwan population-based study revealed that women with BD had a greater risk of breast cancer than did the general population (SIR 2.2, 95% CI 1.004–4.1) [16]. Our study is the first to show that the risk of some solid cancers, especially cancers of organs that can be involved in BD, is higher in patients with BD than that in the general population.

Contrary to the results for solid cancer, the risk of hematologic cancers was not greater in patients with BD than that in the general population. Many previous studies have reported greater risks of hematological cancers in patients with autoimmune diseases such as RA, SLE, Sjögren’s syndrome, systemic sclerosis, and dermatomyositis [5–9]. Recently, two Taiwanese population-based studies also demonstrated that patients with BD had a greater risk of hematological malignancies (SIR, 4.2), especially non-Hodgkin’s lymphoma (SIR, 6.2–8.3) [16,17]. Immune-mediated diseases and hematological cancers have similar genetic susceptibilities and environmental triggers (e.g., Epstein-Barr virus infection) [18], and they also share a common pathogenesis. Several inflammatory cytokines, including tumor necrosis factor-α (TNF-α), interferon-γ, interleukin (IL)-6, and IL-8, play a role in the pathogenesis of both immune-mediated diseases and hematologic cancers [19–22]. These shared factors are thought to be involved in the development of hematologic cancers in patients with immune-mediated diseases. However, contrary to our expectation, only one of 2313 patients with BD in the present study developed hematological cancer (multiple myeloma), and BD was not significantly associated with a greater risk of hematologic cancer. The main reason for this unexpected result may be that the follow-up period in the present study was too short (median, 2.34 years). In the future, long-term studies are needed to clarify the causal relationship between BD and hematological cancer.

However, based on our results, it is difficult to conclude that BD does not increase the risk of hematological cancer because the risk of MDS, which can progress to acute myeloid leukemia [23], was significantly greater in patients with BD than that in the general population. Moreover, the risk of MDS was much greater in comparison to that of other significant solid cancers (SIR, 65.7 in men and SIR, 53.9 in women). Several previous studies have also reported that BD is associated with MDS [10–13]. Furthermore, a review of the literature reported that MDS is markedly more common than leukemia is in Japanese BD patients [12]. Cytogenetic aberrations, especially trisomy 8, are thought to play an important role in the pathogenesis of BD associated with MDS. Of the cytogenetic abnormalities, trisomy 8 has been reported to be most common, occurring in 64–87% of patients with BD associated with MDS [12,13]. However, all of the previous studies regarding BD associated with MDS were case reports or hospital-based studies. Our study is meaningful in that it is the first population-based study to show that BD increases the risk of MDS.

In the present study, the use of thiopurines was associated with lower solid and overall cancer risk in patients with BD. Our results may be in accord with findings of studies of patients with inflammatory bowel disease (IBD). Some studies have shown that immunomodulators reduce the risk of colorectal cancer by suppressing intestinal inflammation in patients with IBD [24,25]. Similar to patients with IBD, immunomodulators may reduce the risk of solid cancer by suppressing the inflammation of the affected organ in patients with BD. Several other studies have reported that immunomodulators may increase the risk of hematological cancers [26]. However, we could not assess the relationship between the use of thiopurines and the risk of hematological cancers because only one patient developed hematological cancer. In our study, the follow-up duration was too short to thoroughly evaluate the effect of medication use on risk of cancer. Future extended, long-term studies are required to elucidate the effects of use of medication including thiopurines and TNF-α inhibitors on cancer development.

Our study had several limitations. First, we did not verify the diagnostic accuracy of BD. As we used insurance claims data, it is possible that some patients with BD were missed or that patients without BD had been misdiagnosed. To overcome this limitation, we only included cases that met the RID code as well as the BD diagnostic code. Given that patients can be registered in the RID system only if they meet strict diagnostic criteria, BD is not likely to be misdiagnosed. In addition, given the medical benefits provided by the RID program, a diagnosis of BD is not likely to be missed. Therefore, we believe that the definition of BD diagnosis in the present study was reliable. Second, since we used administrative data, we could not obtain information on symptoms or signs of BD and the organs that were involved. For example, patients with intestinal involvement may have a greater risk of CRC and patients with neurological involvement may have a greater risk of brain/CNS cancer. However, we could not evaluate the cancer risk according to the involved organs. Third, the duration of follow-up was too short to assess completely the development of BD-associated cancers. We could evaluate cancer risk in the early stages of the disease, but not the long-term cancer risk. Nevertheless, our study showed that patients with BD had an increased risk of overall cancer, solid cancers, and MDS in the early stages of the disease. Many patients with BD may have symptoms of BD, such as recurrent oral ulcers, before an objective diagnosis is made. Additionally, patients with a predisposition toward developing BD may have dysregulated immune responses even before the diagnosis of BD is made. These may result in a high risk of cancer even in the early stage of the disease. Finally, there might have been a detection bias. Patients with BD are more likely to seek health care and to need multiple hospital visits, as compared to the general population, and thus they may have a greater rate of cancer detection than the general population does.

In conclusion, both men and women with BD had a greater risk of overall cancer, some solid cancers (especially in organs that can be involved in BD), and MDS than the general population did. However, the risk of hematological cancer in patients with BD was not significantly greater than that in the general population. Further long-term cohort studies are required to clarify the influence of BD itself, and medications used for its treatment, on cancer development.

## References

[1] Cheon JH, Kim WH. An update on the diagnosis, treatment, and prognosis of intestinal Behçet’s disease. Curr Opin Rheumatol. 2015;27: 24–31. doi: 10.1097/BOR.0000000000000125 25405821 10.1097/BOR.0000000000000125

[2] Dalvi SR, Yildirim R, Yazici Y. Behcet’s Syndrome. Drugs. 2012;72: 2223–2241. doi: 10.2165/11641370-000000000-00000 23153327 10.2165/11641370-000000000-00000

[3] Beyaert R, Beaugerie L, Van Assche G, Brochez L, Renauld JC, Viguier M, et al Cancer risk in immune-mediated inflammatory diseases (IMID). Mol Cancer. 2013;12: 98 doi: 10.1186/1476-4598-12-98 23987103 10.1186/1476-4598-12-98 PMC3765952

[4] Okada F. Inflammation-related carcinogenesis: current findings in epidemiological trends, causes and mechanisms. Yonago Acta Med. 2014;57: 65–72. 25324587 PMC4198572

[5] Turesson C, Matteson EL. Malignancy as a comorbidity in rheumatic diseases. Rheumatology (Oxford). 2013;52: 5–14. 22829694 10.1093/rheumatology/kes189

[6] Weng MY, Huang YT, Liu MF, Lu TH. Incidence of cancer in a nationwide population cohort of 7852 patients with primary Sjogren’s syndrome in Taiwan. Ann Rheum Dis. 2012;71: 524–527. doi: 10.1136/annrheumdis-2011-200402 22072014 10.1136/annrheumdis-2011-200402

[7] Chen YJ, Chang YT, Wang CB, Wu CY. The risk of cancer in patients with rheumatoid arthritis: a nationwide cohort study in Taiwan. Arthritis Rheum. 2011;63: 352–358. doi: 10.1002/art.30134 21279991 10.1002/art.30134

[8] Liang JA, Sun LM, Yeh JJ, Lin WY, Chang SN, Sung HC, et al Malignancies associated with systemic lupus erythematosus in Taiwan: a nationwide population-based cohort study. Rheumatol Int. 2012;32: 773–778. doi: 10.1007/s00296-010-1684-y 21193991 10.1007/s00296-010-1684-y

[9] Kuo CF, See LC, Yu KH, Chou IJ, Chang HC, Chiou MJ, et al Incidence, cancer risk and mortality of dermatomyositis and polymyositis in Taiwan: a nationwide population study. Br J Dermatol. 2011;165: 1273–1279. doi: 10.1111/j.1365-2133.2011.10595.x 21895620 10.1111/j.1365-2133.2011.10595.x

[10] Lin Y, Li G, Zheng W, Tian X, Zhang F. Behcet’s disease associated with malignancy: a report of 41 Chinese cases. Int J Rheum Dis. 2014;17: 459–465. doi: 10.1111/1756-185X.12269 24354961 10.1111/1756-185X.12269

[11] Ahn JK, Oh JM, Lee J, Koh EM, Cha HS. Behcet’s disease associated with malignancy in Korea: a single center experience. Rheumatol Int. 2010;30: 831–835. doi: 10.1007/s00296-009-1319-3 20016987 10.1007/s00296-009-1319-3

[12] Tada Y, Koarada S, Haruta Y, Mitamura M, Ohta A, Nagasawa K. The association of Behcet’s disease with myelodysplastic syndrome in Japan: a review of the literature. Clin Exp Rheumatol. 2006;24: S115–119. 17067441

[13] Ahn JK, Cha HS, Koh EM, Kim SH, Kim YG, Lee CK, et al Behcet’s disease associated with bone marrow failure in Korean patients: clinical characteristics and the association of intestinal ulceration and trisomy 8. Rheumatology (Oxford). 2008;47: 1228–1230. 18550640 10.1093/rheumatology/ken162

[14] Na SY, Shin J, Lee ES. Morbidity of solid cancer in Behcet’s disease: analysis of 11 cases in a series of 506 patients. Yonsei Med J. 2013;54: 895–901. doi: 10.3349/ymj.2013.54.4.895 23709423 10.3349/ymj.2013.54.4.895 PMC3663239

[15] Korea Central Cancer Registry, National Cancer Center. Annual report of cancer statistics in Korea in 2013, Ministry of Health and Welfare, 2015.

[16] Wang LH, Wang WM, Hsu SM, Lin SH, Shieh CC. Risk of Overall and Site-specific Cancers in Behcet Disease: A Nationwide Population-based Study in Taiwan. J Rheumatol. 2015;42: 879–884. doi: 10.3899/jrheum.140770 25834207 10.3899/jrheum.140770

[17] Yu KH, Kuo CF, Huang LH, Huang WK, See LC. Cancer Risk in Patients With Inflammatory Systemic Autoimmune Rheumatic Diseases: A Nationwide Population-Based Dynamic Cohort Study in Taiwan. Medicine (Baltimore). 2016;95: e3540. 27149461 10.1097/MD.0000000000003540 PMC4863778

[18] Baecklund E, Smedby KE, Sutton LA, Askling J, Rosenquist R. Lymphoma development in patients with autoimmune and inflammatory disorders—what are the driving forces? Semin Cancer Biol. 2014;24: 61–70. doi: 10.1016/j.semcancer.2013.12.001 24333759 10.1016/j.semcancer.2013.12.001

[19] Hamzaoui K, Hamzaoui A, Guemira F, Bessioud M, Hamza M, Ayed K. Cytokine profile in Behcet’s disease patients. Relationship with disease activity. Scand J Rheumatol. 2002;31: 205–210. 12369651 10.1080/030097402320318387

[20] Bardak Y, Aridogan BC. The demonstration of serum interleukin 6–8, tumor necrosis factor-alpha, complement, and immunoglobulin levels in Behcet’s disease with ocular involvement. Ocul Immunol Inflamm. 2004;12: 53–58. 15209464 10.1076/ocii.12.1.53.28062

[21] Voulgarelis M, Giannouli S, Ritis K, Tzioufas AG. Myelodysplasia-associated autoimmunity: clinical and pathophysiologic concepts. Eur J Clin Invest. 2004;34: 690–700. doi: 10.1111/j.1365-2362.2004.01417.x 15473894 10.1111/j.1365-2362.2004.01417.x

[22] Hsu HC, Lee YM, Tsai WH, Jiang ML, Ho CH, Ho CK, et al Circulating levels of thrombopoietic and inflammatory cytokines in patients with acute myeloblastic leukemia and myelodysplastic syndrome. Oncology. 2002;63: 64–69. doi: 10.1159/000065722 12187073 10.1159/000065722

[23] Gangat N, Patnaik MM, Tefferi A. Myelodysplastic syndromes: Contemporary review and how we treat. Am J Hematol. 2016;91: 76–89. doi: 10.1002/ajh.24253 26769228 10.1002/ajh.24253

[24] Andersen NN, Jess T. Has the risk of colorectal cancer in inflammatory bowel disease decreased? World J Gastroenterol. 2013;19: 7561–7568. doi: 10.3748/wjg.v19.i43.7561 24282346 10.3748/wjg.v19.i43.7561 PMC3837254

[25] Jess T, Simonsen J, Jorgensen KT, Pedersen BV, Nielsen NM, Frisch M. Decreasing risk of colorectal cancer in patients with inflammatory bowel disease over 30 years. Gastroenterology. 2012;143: 375–381.e371; quiz e313-374. doi: 10.1053/j.gastro.2012.04.016 22522090 10.1053/j.gastro.2012.04.016

[26] Kotlyar DS, Lewis JD, Beaugerie L, Tierney A, Brensinger CM, Gisbert JP, et al Risk of lymphoma in patients with inflammatory bowel disease treated with azathioprine and 6-mercaptopurine: a meta-analysis. Clin Gastroenterol Hepatol. 2015;13: 847–858.e844; quiz e848-850. doi: 10.1016/j.cgh.2014.05.015 24879926 10.1016/j.cgh.2014.05.015

